# mGem: A perfect storm in the era of global warming—the convergence between thermotolerant fungi and altered immunity

**DOI:** 10.1128/mbio.00750-26

**Published:** 2026-07-01

**Authors:** Frederic Lamoth, Dimitrios P. Kontoyiannis

**Affiliations:** 1 Infectious Diseases Service, Department of Medicine, Lausanne University Hospital and University of Lausanne 27213, Lausanne, Switzerland; 2 Institute of Microbiology, Department of Laboratory Medicine and Pathology, Lausanne University Hospital and University of Lausanne 30635, Lausanne, Switzerland; 3 Department of Infectious Diseases, Infection Control and Employee Health, The University of Texas MD Anderson Cancer Center 4002, Houston, Texas, USA; Vallabhbhai Patel Chest Institute, Delhi, India

**Keywords:** climate, natural disasters, pollution, molds

## Abstract

The evidence supporting the impact of global warming on the epidemiology of infectious diseases, including fungal infections, is increasing. Fungi have a remarkable ability to adapt to heat and pollution, and to disseminate via air, water ecosystems, or wildfire smoke. Their genetic plasticity can lead to thermotolerance, the ability to find new ecological niches, and fitness gains. Natural reservoirs of the fungal biomass, which are heavily affected by global warming, may serve as the environments from where new fungal diseases originate, as illustrated by the recent emergence of *Candidozyma auris* and *Rhodosporidiobolus fluvialis*. Moreover, global warming also affects human skin/mucosal integrity and local or systemic immune responses, which could increase host susceptibility to fungal infections. This review examines the impact of global warming on the complex fungi-host interactions, which can lead to new challenges in mycology, and discusses possible mitigation strategies.

## PERSPECTIVE

The accelerated effect of global warming on Earth is one of the most important challenges in our century. Consequences of climate change are multiple, including progressive alterations of ecosystems and increases in the frequency of natural disasters, which may lead to the emergence of new pathogens and the expansion of the epidemiological footprint of existing pathogens. For humans, adaptive mechanisms to global warming that may affect our vulnerability to infections are both societal (migration, famine or poor sanitary conditions resulting from natural disasters, changes in industry/agriculture practices, and alterations in lifestyle), environmental (loss of biodiversity in land and aquatic systems, expansion of the geographic range of vectors and changes in their ecology, changes in migratory patterns of birds), and biological (modifications of microbiota and changes in innate and adaptive immune responses). While emerging waterborne or arthropod-associated viral (e.g., dengue) or bacterial diseases (e.g., enteric bacterial pathogens) have drawn most attention, there is increasing awareness of the role of fungi (1–4). A systematic search found 24 examples of fungal diseases with some link to climate change hazards, which represents about 10% of all impacted infectious diseases (5). This may only represent the tip of the iceberg considering the rich diversity and environmental adaptability of fungal species and the spectrum of diseases they cause. In the animal kingdom, we had a recent example of the potential of fungi in taking advantage of global warming and profoundly disturbing living ecosystems with the emergence of the fungus *Batrachochytrium dendrobatidis* leading to mass extinction of amphibian species (6). In this review, we will discuss the potential of fungi to thrive in a warming planet, and the preventive strategies we could employ to meet this new public health threat.

## HOW FUNGI SEIZE THE MOMENT IN A WARMING PLANET

Fungi are ubiquitous in the environment, are the most important catalysts in the decomposition of organic matter, and represent a substantial part of the biomass (7). They are common symbionts on mucosal surfaces of humans. Indeed, superficial fungal diseases of the skin or mucosa have been known since antiquity and remain an important cause of morbidity worldwide (8). Moreover, fungi can take advantage of a compromised immune system, as illustrated by the emergence of invasive fungal diseases during the second half of the 20th century as a consequence of the progress of medicine and the expanding immunocompromised population with longer life expectancy (9). Of interest, typical opportunist molds (*Aspergillus*, *Mucorales*) have emerged as causes of secondary lung infections following severe viral illnesses such as influenza or SARS-CoV-2 (10). Increasing awareness and epidemiological surveys, as well as improved diagnostic tools, may have also contributed to a better recognition of fungal infections. However, increasing evidence suggests that global warming may create a new niche for some fungi and remodel the epidemiology of fungal diseases (4).

Fungi have the potential for thermal adaptation, as well as thermally-induced morphogenetic changes (i.e., switch from hyphal to yeast form). A recent study showed that fungal isolates from warmer neighborhoods of the heat island of Baltimore City exhibited better thermotolerance and lighter pigmentation compared with isolates from colder suburban areas (11). Adaptive thermotolerance could reveal the potential virulence of some currently non-pathogenic species if they become able to overcome the thermal barrier of endothermic mammals, leading to the emergence of novel fungal diseases or epidemiological expansion of existing fungal pathogens (12–14). A possible example of thermal selection is the emergence of *Candidozyma auris*, which was first recognized in 2009 and spread on all continents with genotypically distinct clades suggesting an evolution from a common ancestor and a shared underlying trigger, such as global warming (15, 16). *C. auris* was recovered in wetlands of temperate regions (e.g., mangroves, estuaries) (17, 18). It grew optimally at higher temperatures (42°C) compared with other pathogenic yeasts and also exhibits high salt tolerance (19). Phylogenetic studies suggested that the clades have diverged only a few hundred years ago (20), which suggests a recent evolutionary event. However, many questions remain open about *C. auris*, such as its natural habitat and the way of transmission between humans and the environment, and triggers other than global warming may have contributed to its emergence.

Another novel fungal pathogen, *Rhodosporidiobolus fluvialis*, has been recently reported as a cause of fungemia in two patients in China (21). This yeast exhibits temperature-dependent dimorphism (yeasts to pseudohyphae), resulting in hypervirulence and multi-drug resistance (21). Similarly to *C. auris*, *R. fluvialis* was initially discovered from marine environments, highlighting the likely role of these ecosystems as a reservoir of novel fungal pathogens (22). In particular, mangroves, which retain organic matter, host large and diverse fungal communities, including known pathogenic species (23). Stagnation of mixed fresh and saline water in mangroves represents an ideal culture broth for the selection of fungi under warming conditions. Fungi can adapt to this environment because of their ability to forage for nutrients, resist heat or salinity, and detoxify pollutants (e.g., heavy metals) (24). The role of environmental pollution as a factor for the emergence of fungal pathogens is still underinvestigated. A study reported the consequences of contamination of tropical rivers on fungal diversity and the proliferation of potentially pathogenic fungal species (25). Moreover, marine biomass remains largely unknown, with an estimated proportion of 90% of fungi that still remain to be discovered and represent potential novel pathogens (26, 27).

Similarly, pollution and global warming could be drivers of increased incidence of a variety of diseases caused by airborne fungi. Molds are capable of disseminating via spores, which are resistant to heat and other environmental stresses (e.g., pollutants). Changes in air quality associated with global warming (pollution and increase in allergen concentrations due to extension of flowering seasons) may impact the epidemiology of airborne mold infections and increase the burden of fungal allergic respiratory diseases (28, 29). Desertification may be associated with a decrease in pollen propagation but could result in increasing concentration of airborne particulate matter, which could damage airways and increase susceptibility to infection. Wildfire activity and megafires have increased exponentially as climate change progresses. Fungal spores are abundant in fire smoke and can be transported over long distances (30, 31). Moreover, wildfires have a considerable impact on the fungal biomass with a decrease of its richness and redistribution of fungal species over time, resulting in a predominance of pathogenic Ascomycota (32). In particular, these conditions may favor *Aspergillus* spp., which have heat-resistant spores, can germinate at high temperature, and use various carbon and nitrogen sources from pyrogenic organic matter (33). Wildfire smoke exposure has been associated with a significant increase of hospital admission for coccidioidomycosis in California (34). Other natural disasters generating large amounts of dust or waterborne contamination (e.g., hurricanes, tornadoes, volcanic eruptions, floods, tsunamis) have been associated with clusters of mold infections, notably dermatophytoses and cutaneous mucormycosis (1, 35–38). An association of thunderstorm days with increased fungal spore concentrations in air and hospital admission for asthma has been demonstrated (39). The long-term downstream effects of such events are largely unknown. For example, fungal sensitization has been implicated in worsening preexisting structural lung diseases, such as bronchiectasis (40).

Some fungal species may also extend their geographical distribution. Recent mathematical modeling suggests that pathogenic *Aspergillus* spp., in particular *A. flavus* and *A. niger*, more adapted to warm regions, may shift their habitat suitability to northern regions (41). Trends of geographical expansion are observed for histoplasmosis and other endemic mycoses (42–45). Climate projections suggest that cases of coccidioidomycosis in the United States will increase by 50% by 2100 because of the extension of regions with dry and warm climate (46). Sporotrichosis is increasingly reported from low-endemic areas (e.g., Southern Europe), and a novel virulent species (*Sporothrix brasiliensis*) is spreading in South America (47–49). Interestingly, *S. brasiliensis* is essentially transmitted by cats and is adapted to the relatively high body temperature of this mammal (50).

The causes of these epidemiological changes often remain unclear and may result not only from global warming itself but also from indirect effects of climate changes, such as natural disasters. For instance, *Cryptococcus gattii* has expanded in temperate areas, such as the Mediterranean basin, but its establishment in relatively cold climatic regions, such as the Northwest American coasts, remains enigmatic and could have resulted from shipborne transport and the late effect from the 1964 tsunami (51, 52).

Finally, various fungi causing subcutaneous infections (mycetoma, chromoblastomycosis) can be transmitted via contaminated plants or by insect bites. In Ethiopia’s Asfar region, one-third of mycetoma cases have been associated with thorn injury from *Prosopis julifora*, a climate-resistant tree that has been implanted to combat desertification (53). It is also speculated that global warming could increase the density, geographic range, and aggressive behavior of insects that are vectors of many fungi causing cutaneous or subcutaneous mycoses, although there is currently no evidence of increasing incidence of such infections (54).

A summary of evidence supporting the impact of global warming and its consequences on the epidemiology of fungal diseases is provided in Table 1.

**TABLE 1:** Direct and indirect impact of climate changes on the epidemiology of pathogenic fungi in humans

Trigger	Fungi	Observed or putative effects (references)
Change in climatic conditions (increased temperature, dryness or humidity)	*Coccidioides immitis*	Increased incidence in endemic areas (43, 55), expansion of geographical areas (43, 46, 56, 57)
	*Sporothrix* spp.	Expansion of geographical areas (48, 58, 59)
	Other dimorphic fungi (e.g., histoplasmosis, blastomycosis)	Expansion of geographical areas (43)
	*Cryptococcus gattii*	Expansion of geographical areas (51, 60)
	Fungi causing subcutaneous mycoses (e.g., mycetoma, chromoblastomycosis)	Expansion of geographical areas (61)
	*Malassezia* and dermatophytes	Increased incidence (62)
	*Aspergillus* spp.	Expansion of geographical areas for some species and species redistribution (41)
Alteration of aquatic ecosystems (warming, increased salinity)	*Candidozyma auris*	Emergence as fungal pathogen (15, 16, 63)
	*Rhodosporidiobolus fluvialis*	Emergence as fungal pathogen (21)
Natural disasters (tornadoes, hurricanes, flooding, tsunamis)	*Coccidioides immitis*	Increased incidence or large clusters (64–66)
	Dermatophytes	Increased incidence (1, 35)
	Fungi causing subcutaneous mycoses (e.g., mycetoma, chromoblastomycosis)	Increased incidence (67, 68)
	Mucorales	Clusters of cutaneous mucormycosis (36, 37)
	Other invasive molds (e.g., *Aspergillus*)	Increased incidence or clusters of unusual presentation (38, 69, 70)
Better adaptation to/spread of animal vectors (e.g., birds, mammals, sea life)	*Cryptococcus* spp.	Expansion of geographical areas (71)
	*Sporothrix brasiliensis*	Emergence as fungal pathogen (49)
	*Candidozyma auris*	Emergence as fungal pathogen (15)
Proliferation/spread of invertebrate vectors (e.g., arthropods)	Fungi causing mycetoma or other subcutaneous mycoses	Putative increase of incidence or expansion to new geographical areas in future (54)
Changes in vegetation	Fungi causing subcutaneous mycoses (e.g. mycetoma, chromoblastomycosis)	Increased incidence in affected regions (53)
Changes in air quality (e.g., pollution, wildfires, megafires, thunderstorms)	Airborne allergenic fungi (e.g., *Aspergillus* spp., *Cladosporium* spp.)	Increased prevalence of asthma and chronic respiratory diseases (28, 29, 39)
	*Coccidioides immitis*	Increased incidence (34)

## EFFECTS OF GLOBAL WARMING ON HOST VULNERABILITY TO FUNGAL INFECTIONS

Many fungi are opportunistic pathogens, and their pathogenicity does not only rely on their intrinsic virulence but also, and mainly, on the host immune responses, which can be affected by chronic heat stress. There is increasing evidence that heat stress affects the function of vital organs and can disturb innate and adaptive immune responses (72, 73). Activation of heat shock transcription factors (HSFs) and heat shock proteins (HSPs) can increase the production of pro-inflammatory cytokines, such as interleukins (IL-1, IL-4, IL-6), tumor necrosis factor alpha (TNFα), and interferon-gamma (IFN-γ), or anti-inflammatory cytokines (IL-10), disrupting homeostasis and possibly weakening antifungal immune responses (73–76). For instance, IL-6, which is induced by heat stress, has been associated with worse outcomes of invasive aspergillosis (77, 78).

In addition, temperature variation, humidity, ultraviolet radiation, and air pollution may also affect the natural barriers against fungal infections. Their impact on the skin and mucosa barrier is the most studied. Activation of heat sensors in the skin (e.g., transient receptor potential vanilloid 3, TRPV3) may result in chronic skin inflammation (79). Small particulate matters contained in pollutants may disturb the skin or mucosa epithelium via degradation of keratinocyte tight junctions and upregulation of genes encoding for cholesterol production with subsequent alteration of sebum (80, 81). Although the role of altered skin barrier could not be demonstrated, some cutaneous/subcutaneous mycoses are more frequent in warmer climatic conditions (82). For instance, increased incidence of tinea and pityriasis versicolor has been observed in association with the El Niño wave (62). *C. auris* is primarily a skin colonizer of moist body areas, and global warming might favor colonization and transmission in the future. Moreover, direct or indirect effects of climate changes may also affect the skin microbiome and favor various skin disorders, although the impact of such alterations on cutaneous mycoses is unclear (83).

High temperature also affects lung epithelium, triggering inflammation and bronchial hyperreactivity (79). Water evaporation and air dryness, which is a hallmark of global warming, have been associated with a thinner mucus layer and increasing inflammatory cytokine production (TNFα, IL-33, IL-6) (84). Indirect effects of climate changes, such as pollutants and wildfire smoke, impact respiratory health with increased burden of asthma and chronic obstructive pulmonary disease (COPD) (85, 86). In particular, wildfires have been associated with worse asthma outcomes (87, 88). Asthma and COPD may favor the development of allergic bronchopulmonary aspergillosis, chronic or invasive aspergillosis (89–91).

The effect of warming on the intestinal barrier has been less studied. An *in vitro* model showed that small particulate matters can induce intestinal inflammation through oxidative stress and impaired T cell/T helper cell 17 regulation (92, 93). Whether this may increase the risk of intra-abdominal mycoses, such as candidiasis, is unknown.

Pollution may also affect innate immune memory and immunotolerance, which can alter susceptibility to infection (94). Finally, heat may favor underlying conditions that are associated with an increased risk of invasive fungal infections. Notably, patients with diabetes mellitus are at increased risk of complications, including diabetic ketoacidosis, because of dehydration, dysautonomia, and impaired thermoregulation during heat waves (95, 96). Decompensated diabetes and ketoacidosis are well-known predisposing factors for mucormycosis and possibly other invasive fungal infections (such as invasive candidiasis, aspergillosis, or fusariosis) (97–100). Other effects of global warming (e.g., air pollution, humidity) have also been associated with increased risk of autoimmune diseases (rheumatoid arthritis, sarcoidosis) and COPD (101–103).

**Fig 1:**
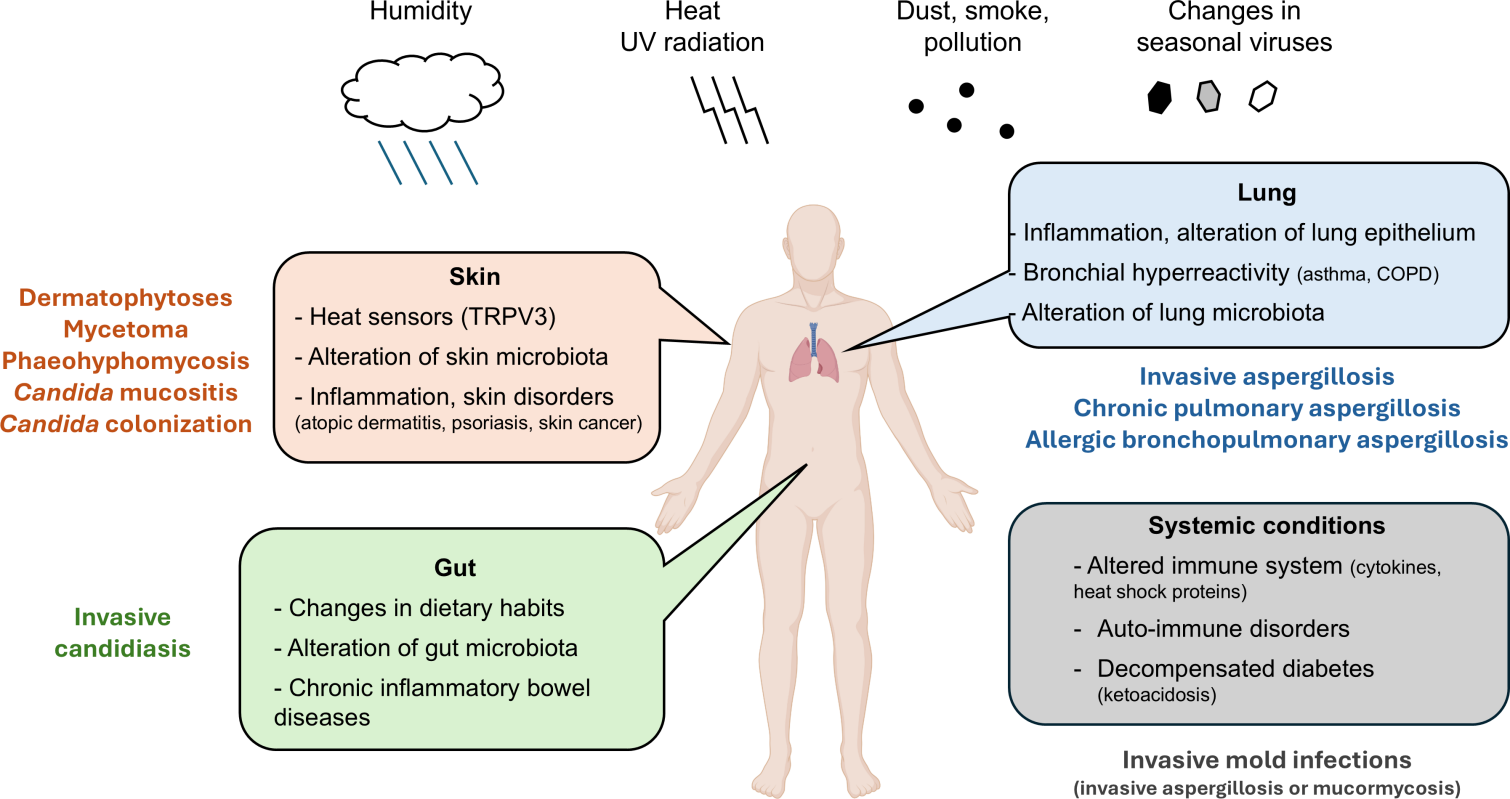
Impact of global warming and its indirect consequences on the human immune system and susceptibility to fungal diseases. Immune alterations for different systems (skin, gut, lung, systemic conditions) are represented in boxes with their corresponding fungal diseases (free text out of boxes of same color). UV: ultraviolet, TRPV3: transient receptor potential vanilloid 3, COPD: chronic obstructive pulmonary disease. Image generated using BioRender.

The diverse impact of altered host susceptibility on fungal diseases is summarized in Fig. 1.

## FUTURE PREVENTIVE AND CONTROL STRATEGIES

The rapid evolution of fungal epidemiology, which may partly result from climate changes and their multiple consequences, warrants increasing attention. Early detection of new fungal pathogens is crucial. As microbiology laboratories may fail to identify fungi that exhibit poor growth in culture or are not detected by conventional diagnostic tools, culture-independent molecular tools (targeted PCRs, metagenomics) are important. Genomic surveillance with whole genome sequencing is also necessary to identify distinct lineages, which may help identify the sources and transmission routes, as well as detect new virulence or resistance traits.

Detection of clusters of unusual fungal infections should be reported to national public health authorities and trigger investigations into the sources and epidemiological trends. International collaborative networks should be set up to implement surveillance programs and get prepared for managing outbreaks. The effort of the World Health Organization (WHO) to establish a fungal priority pathogens list represents an important step to coordinate global public health action (104). Successful investigation will rely on the ability to perform large clinical and environmental sample collection with genotyping analyses. This approach should also integrate data from other parameters associated with global warming, such as meteorological and demographic data, analyses of air or water composition, and search for potential zoonotic modes of transmission (105). Measures should be taken to decrease the effect of heat islands in cities and their impact on fungal thermal adaptation and human physiology/immunity (11). Predictive models, using mathematical modeling and computational analyses, could anticipate future evolution, as it has been done for coccidioidomycosis, malaria, or cholera (46, 106–108). The rapid development of artificial intelligence is expected to improve such strategies.

Finally, it should be emphasized that, despite increasing awareness of the impact of global warming on human health, international collaborations and implementation of concrete measures by competing authorities remain difficult to achieve.
